# 2174. Cefiderocol Activity Against Multidrug-resistant and Molecularly Characterized *Pseudomonas aeruginosa* and *Acinetobacter baumannii-calcoaceticus* complex Clinical Isolates Causing Infection in United States Hospitals (2020–2022)

**DOI:** 10.1093/ofid/ofad500.1796

**Published:** 2023-11-27

**Authors:** Rodrigo E Mendes, Cory Hubler, Valerie Kantro, Dee Shortridge, Helio S Sader, Mariana Castanheira

**Affiliations:** JMI Laboratories, North Liberty, Iowa; JMI Laboratories, North Liberty, Iowa; JMI Laboratories, North Liberty, Iowa; JMI Laboratories, North Liberty, Iowa; JMI Laboratories, North Liberty, Iowa; JMI Laboratories, North Liberty, Iowa

## Abstract

**Background:**

Cefiderocol (CFDC) is a siderophore-conjugated cephalosporin with activity against Gram-negative bacteria. CFDC and comparator activities were evaluated against resistant and molecularly characterized *P. aeruginosa* (PSA) and *A. baumannii-calcoaceticus* complex (ABC) as part of the SENTRY Antimicrobial Surveillance Program.

**Figure:**
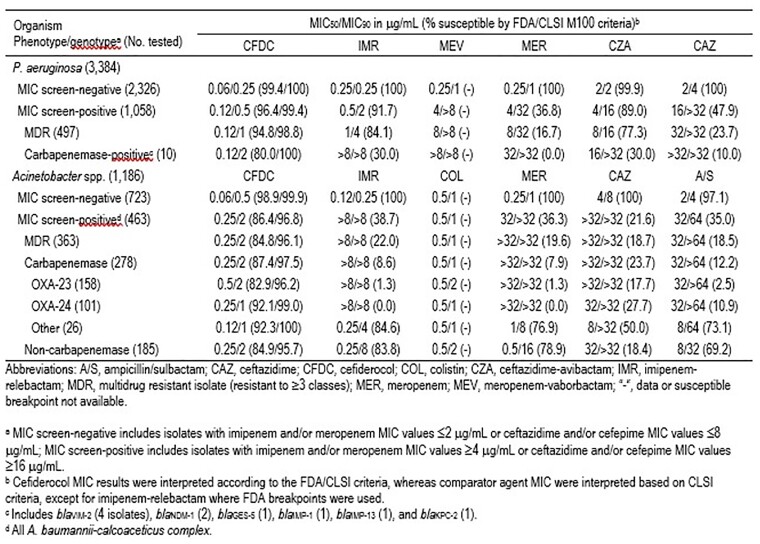

**Methods:**

3,384 PSA and 1,186 *Acinetobacter* spp. (979 ABC) were consecutively collected from 64 US sites in 2020–2022. Susceptibility testing was performed by broth microdilution and CFDC testing used iron-depleted media. FDA and CLSI breakpoints were used for CFDC. CLSI criteria were applied to comparators, except for imipenem-relebactam (IMR; FDA). Isolates with a resistance phenotype to ≥ 3 classes were defined as multidrug resistant (MDR). ABC and PSA with imipenem and/or meropenem (MER) MICs ≥ 4 μg/mL or ceftazidime (CAZ) and/or cefepime MICs ≥ 16 μg/mL were subjected to genome sequencing for screening of acquired carbapenemase genes.

**Results:**

31.3% (1,058/3,384) and 14.7% (497/3,384) of PSA met the MIC screening criteria for molecular characterization and showed an MDR phenotype, respectively (Table). Carbapenemase genes were detected in 10 PSA (< 1%). CFDC had similar MICs against PSA (MIC_50/90_, 0.12/0.5 μg/mL) that did meet the MIC screening criteria and against those that did not (MIC_50/90_, 0.06/0.25 μg/mL). CFDC also had similar MIC_50_ values (0.12 μg/mL) against the MDR and carbapenemase-positive PSA populations, whereas other agents had compromised activity. 47.3% (463/979) of ABC met the MIC screening criteria, while 37.1% (363/979) had an MDR phenotype, and 28.4% carried carbapenemase genes. In general, CFDC, IMR, meropenem-vaborbactam, colistin (COL), MER, ceftazidime, and CAZ had activity against ABC that did not meet the MIC screening criteria, but only CFDC (MIC_50/90_, 0.12–0.5/1–2 μg/mL) and COL were active against the resistant ABC subsets.

**Conclusion:**

Many PSA had a MDR phenotype but acquired carbapenemase genes remained rare in this subset. Resistance and presence of carbapenemase genes were high in ABC. CFDC showed potent activity against PSA and ABC subsets in US hospitals, including across resistant and molecularly characterized subsets, where treatment options are limited.

**Disclosures:**

**Rodrigo E. Mendes, PhD**, AbbVie: Grant/Research Support|Basilea: Grant/Research Support|Cipla: Grant/Research Support|Entasis: Grant/Research Support|GSK: Grant/Research Support|Paratek: Grant/Research Support|Pfizer: Grant/Research Support|Shionogi: Grant/Research Support **Cory Hubler, BS**, AbbVie: Grant/Research Support|Shionogi: Grant/Research Support **Valerie Kantro, BA**, AbbVie: Grant/Research Support|Pfizer: Grant/Research Support|Shionogi: Grant/Research Support **Dee Shortridge, PhD**, Melinta: Grant/Research Support|Shionogi: Grant/Research Support **Helio S. Sader, MD, PhD, FIDSA**, AbbVie: Grant/Research Support|Basilea: Grant/Research Support|Cipla: Grant/Research Support|Paratek: Grant/Research Support|Pfizer: Grant/Research Support|Shionogi: Grant/Research Support **Mariana Castanheira, PhD**, AbbVie: Grant/Research Support|Basilea: Grant/Research Support|bioMerieux: Grant/Research Support|Cipla: Grant/Research Support|CorMedix: Grant/Research Support|Entasis: Grant/Research Support|Melinta: Grant/Research Support|Paratek: Grant/Research Support|Pfizer: Grant/Research Support|Shionogi: Grant/Research Support

